# Inorganic Nanoparticles and Composite Films for Antimicrobial Therapies

**DOI:** 10.3390/ijms22094595

**Published:** 2021-04-27

**Authors:** Vera Alexandra Spirescu, Cristina Chircov, Alexandru Mihai Grumezescu, Bogdan Ștefan Vasile, Ecaterina Andronescu

**Affiliations:** 1 Department of Science and Engineering of Oxide Materials and Nanomaterials, University Politehnica of Bucharest, 011061 Bucharest, Romania; veraspirescu@gmail.com (V.A.S.); cristina.chircov@yahoo.com (C.C.); bogdan.vasile@upb.ro (B.Ș.V.); ecaterina.andronescu@upb.ro (E.A.); 2 Research Institute of the University of Bucharest—ICUB, University of Bucharest, 050657 Bucharest, Romania

**Keywords:** inorganic nanoparticles, antimicrobial therapy, nanobiocides, gold nanoparticles, silver nanoparticles, copper nanoparticles, zinc oxide nanoparticles, titanium oxide nanoparticles, magnesium oxide nanoparticles, iron oxide nanoparticles

## Abstract

The development of drug-resistant microorganisms has become a critical issue for modern medicine and drug discovery and development with severe socio-economic and ecological implications. Since standard and conventional treatment options are generally inefficient, leading to infection persistence and spreading, novel strategies are fundamentally necessary in order to avoid serious global health problems. In this regard, both metal and metal oxide nanoparticles (NPs) demonstrated increased effectiveness as nanobiocides due to intrinsic antimicrobial properties and as nanocarriers for antimicrobial drugs. Among them, gold, silver, copper, zinc oxide, titanium oxide, magnesium oxide, and iron oxide NPs are the most preferred, owing to their proven antimicrobial mechanisms and bio/cytocompatibility. Furthermore, inorganic NPs can be incorporated or attached to organic/inorganic films, thus broadening their application within implant or catheter coatings and wound dressings. In this context, this paper aims to provide an up-to-date overview of the most recent studies investigating inorganic NPs and their integration into composite films designed for antimicrobial therapies.

## 1. Introduction

Nanotechnology involves the controlled manipulation of matter at the nanoscale in order to develop functional materials and structures using a variety of chemical and/or physical methods [1,2]. In other words, nanotechnology can be defined based on the requirements imposed by the US National Nanotechnology Initiative, namely, (i) the development of technology at atomic, molecular, or macromolecular levels within the 1–100 nm range; (ii) the creation and application of structures, devices, and systems with novel properties and functions due to small sizes; and (iii) control or manipulation at the atomic or molecular scale [1]. Owing to the continuous progress and advancements, nanotechnology has become an essential component of everyday life, with many online repositories, such as Nanodatabase, Nanowerk, and StatNano, listing thousands of commercially fabricated nanotechnology products [3]. The wide application of nanomaterials stems from the dramatic improvements of their chemical, physical, mechanical, and optical properties in comparison to bulk materials [1,3,4,5].

Precisely, nanoparticles (NPs), a wide class of nanomaterials that are defined as solid colloidal particles or particulate substances [5,6,7,8,9], are of considerable scientific importance, acting as connecting links between molecular structures and the macromolecular or bulk materials [10]. This interest is demonstrated by the 25-fold increase between 2005 and 2010 in the number of products containing or requiring NPs for their production [11]. The unique and novel features of NPs, including light absorption and scattering, electrical and thermal conductivity, melting point, wettability, and catalytic activity, have been widely explored in many industrial applications [6,8,12,13,14]. Furthermore, the increased surface-to-volume ratio, surface reactivity, charge, shape, and magnetic properties make NPs promising candidates within the biomedical, pharmaceutical, and cosmetic fields [7,11,13,15]. Specifically, NPs allow for attaching various molecules onto their surfaces, thus enabling a variety of applications for medical diagnosis and treatment [12,16].

**Figure 1:**
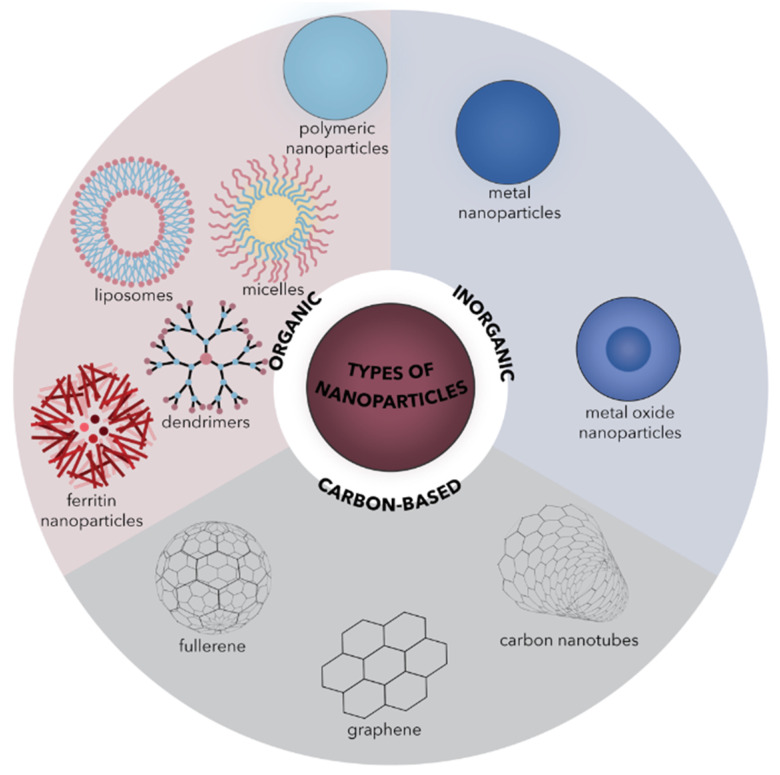
Schematic representation of the main types of NPs classified into organic, inorganic, and carbon-based NPs.

Generally, NPs can be classified into organic, including polymer NPs, dendrimers, micelles, liposomes, lipid-based NPs, and ferritin; inorganic, including metal and metal oxide NPs (e.g., silver, gold, iron oxide, zinc oxide, and silica); and carbon-based NPs, including fullerenes, graphene, and carbon nanotubes (Figure 1) [6,17,18,19,20,21,22,23,24]. While all types of NPs have been intensively studied for drug delivery applications, inorganic NPs provide more suitable features that ensure the protection and bioavailability of the drug with targeted action, limited adverse reactions to the organism, and higher efficiency due to improved drug transport and penetration [6,25,26]. Among the envisaged disease treatments, antimicrobial therapies are of considerable importance due to the possibility of developing alternative antimicrobial agents with the ability to destroy or inhibit the growth of antimicrobial-resistant pathogens [26,27,28,29]. In this regard, inorganic NPs demonstrated increased effectiveness both as nanobiocides due to their intrinsic antimicrobial properties and as nanocarriers for antimicrobial drugs [28]. This is of particular importance, as antimicrobial resistance, which results in standard treatment inefficiency and the subsequent persistence and spreading of infections, has become a global public health emergency [28,30,31,32,33,34,35]. Precisely, according to the available statistics, there are up to 700,000 deaths due to antimicrobial resistance worldwide annually, with the number expected to rise to 10 million deaths by 2050 [28,36].

In order to enhance the efficiency of the inorganic NP-based antimicrobial agents, recent years have witnessed tremendous efforts towards their integration within organic/inorganic components for the development of new and advanced composite materials that combine the advantages of both material types [19,37,38,39,40]. In this manner, composite films comprising inorganic NPs and an organic/inorganic matrix are fabricated and further applied for various antimicrobial therapies, including implant or catheter coatings and wound dressings.

Therefore, the aim of this paper is to provide an up-to-date overview of the most recent studies investigating inorganic NPs and their integration into composite films designed for antimicrobial therapies.

## 2. Inorganic Nanoparticles with Antimicrobial Properties

**Figure 2:**
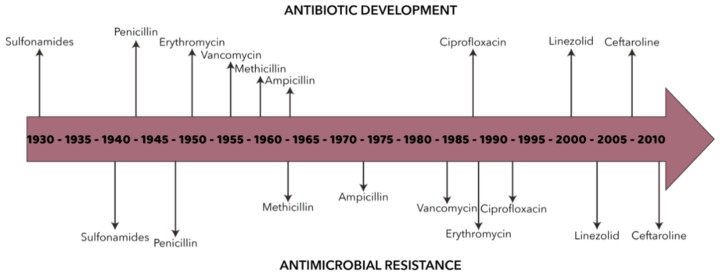
Schematic representation of antibiotic development and antimicrobial resistance appearance over time. Adapted from an open-access source [60,61].

The global health system has been greatly impacted by the discovery of antimicrobials, including antibacterial, antiviral, antifungal, and antiparasitic drugs, which has resulted in a significant reduction in infection-related morbidity and mortality [41,42,43]. However, microorganisms have gradually developed various resistance mechanisms against all types of antimicrobial drugs available on the market (Figure 2). Therefore, it has become a critical issue for modern medicine and drug discovery and development with severe socio-economic and ecological implications [44]. In this context, the term “ESKAPE” has been introduced by the Center for Disease Control and Prevention in order to emphasize the six pathogens that are increasingly escaping the bactericidal effects of antibiotics (i.e., E: *E. faecium*, S: *S. aureus*, K: *K. pneumoniae*, A: *A. baumannii*, P: *P. aeruginosa*, E: E. spp.) [45,46]. These pathogens are generally categorized as multidrug-resistant, extensively drug-resistant, or pan drug-resistant [47], and represent the worldwide leading cause of nosocomial infections [48,49,50,51,52].

**Figure 3:**
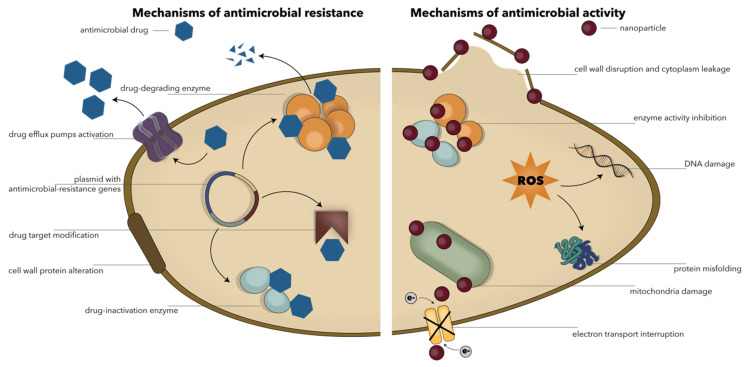
Schematic representation of the main mechanisms involved in the antimicrobial resistance (**left**) and the antimicrobial activity of NPs (**right**). Reprinted from an open-access source [41].

Antimicrobial resistance can be generally classified into natural resistance, either intrinsic (i.e., universally shared within a species and independent of previous exposure to antimicrobial drugs) or induced (i.e., resistance genes naturally occur within the microorganism but are expressed after exposure to the drug), and acquired resistance, which is associated with the acquisition of resistance-related genetic material through transformation, transposition, and conjugation (i.e., horizontal gene transfer) or with mutations to its chromosomal nucleic acid [53,54]. Depending on the biochemical route employed, there are four main types of mechanisms involved in antimicrobial resistance, including (1) decreased drug accumulation through reduced membrane permeability, active extruding of the drug, or increased active drug efflux across the membrane; (2) drug modification or inactivation via enzyme production; (3) drug targeting or binding through site changing and/or bypass; and (4) metabolic pathway alteration (Figure 3) [33,53,55,56,57,58]. Thereby, the malleability and plasticity of the microbial genome lead to a great potential for adaptability and contribute to the development of antimicrobial resistance [59].

In this context, NPs are used as antimicrobial agents either for combatting resistance against antimicrobial drugs themselves or for the delivery of conventional antimicrobials. Their efficiency is generally related to the potential of NPs to penetrate and disrupt the membrane of the microbial cells via membrane-damaging abrasiveness; to decrease the permeability of the cell; to induce antimicrobial effects within the cells, e.g., reactive oxygen species (ROS) production, nucleic acids and protein interactions, enzyme inactivation, and the overexpression of efflux pumps; to release metal ions; and to hinder the formation of biofilms (Figure 3) [20,29,41,62,63,64,65]. Moreover, NPs allow for an improved drug loading and delivery of both hydrophilic and lipophilic molecules as they are able to pass the reticuloendothelial system and internalize the antimicrobials [20].

The antimicrobial potential of NPs is directly influenced by several key factors related to their physico-chemical properties, namely, chemistry, particle size and shape, surface charge and zeta potential, solubility, stability, and the surface-to-volume ratio [20,41,66]. Specifically, cellular biodistribution and uptake are influenced by the surface charge of the NPs, as it drives most of the interactions with tissues and tissue components. Furthermore, hydrophobicity modulates interactions with the phospholipid layer within the microbial membrane, while hydrophilic NPs have longer blood circulation due to reduced interactions with opsonins [20].

**Figure 4:**
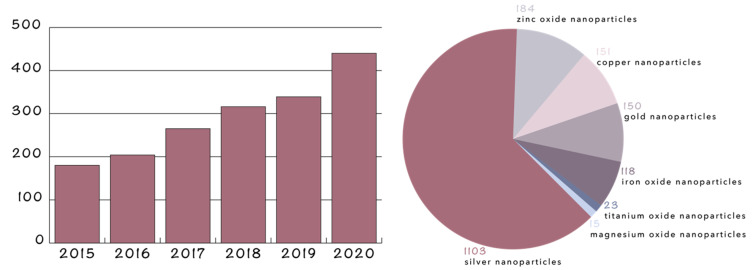
Number of publications between 2015 and 2020 (**left**) and for each type of NPs (**right**) for AuNPs, AgNPs, CuNPs, ZnO NPs, TiO_2_ NPs, MgO NPs, and Fe_3_O_4_ NPs.

**Figure 5:**
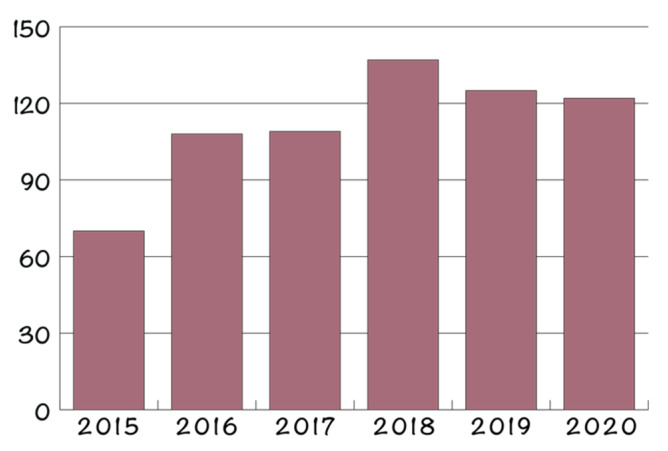
Number of patents between 2015 and 2020 based on therapeutic nanoparticles with antimicrobial activity.

The most commonly used NPs for antimicrobial therapies involve metal NPs, e.g., gold (AuNPs), silver (AgNPs), and copper (CuNPs) [26,27,29]; metal oxide NPs, e.g., zinc oxide (ZnO NPs), titanium oxide (TiO_2_ NPs) [26,27], and magnesium oxide (MgO NPs); and magnetic NPs, e.g., iron oxide NPs (Fe_3_O_4_ NPs) [67,68]. Each type of NP is characterized by specific properties that direct the antimicrobial mechanisms [29]. In this context, Figure 4 depicts the number of original research articles found on the Scopus database using the keywords “antimicrobial activity” and “silver nanoparticles”, “gold nanoparticles”, “copper nanoparticles”, “zinc oxide nanoparticles”, “titanium oxide nanoparticles”, “magnesium oxide nanoparticles”, “iron oxide nanoparticles”, or “magnetite nanoparticles”, without considering research works investigating their incorporation into other components, such as hydrogels, (nano)fibers, films, and scaffolds. Additionally, it should be mentioned that the research articles investigate both the intrinsic antimicrobial properties of NPs and their capacity to deliver antimicrobial drugs to the targeted site. Similarly, Figure 5 depicts the number of patents found on the Scopus database using the keywords “therapeutic nanoparticles” and “antimicrobial activity”.

In this context, Table 1 summarizes the main ideas described in the following paragraphs in terms of synthesis methods and antimicrobial mechanisms for all NP types.

### 2.1. Gold Nanoparticles

AuNPs, also termed as gold colloids, are the most stable among metallic NPs [69,70]. Generally, gold can be found in the Au^+^ (aurous) and Au^3+^ (auric) oxidation states and the Au^0^ non-oxidized state. In the context of NP synthesis, the non-oxidized state is the desirable final state due to its increased stability [71]. Therefore, the two main steps involved in the preparation process involve reduction and stabilization [69]. Specifically, the synthesis of AuNPs involves reducing agents (e.g., citric acid, oxalic acid, hydrogen peroxide, borohydrides, polyols, or sulfites), which act as electron donors that will reduce Au^+^ or Au^3+^ to Au^0^. Subsequently, stabilizing agents (e.g., trisodium citrate dihydrate; thiolates; phosphorus ligands; polymers; or surfactants, such as cetyltrimethylammonium bromide) are added to the reaction in order to prevent aggregation by providing repulsive forces for the control of NP growth in terms of rate, size, and shape [71]. In this context, recent years have witnessed increased attention towards green synthesis methods that use plants and plant parts, fungi, and microorganisms, since their extracts are widely rich in natural reducing and stabilizing agents [72].

While there are many studies demonstrating the intrinsic antibacterial and antifungal properties of AuNPs, their effects are still ambiguous [73,74]. Reports have suggested that the mechanisms involved in the antimicrobial effects of AuNPs include interactions between NPs and the microbial cell wall governed by electrostatic forces and carbohydrate, lipid, and protein binding; damages of the microbial cell membrane and wall and subsequent ribosome and mitochondrion impairment; and inhibition of thiol groups present within bacterial cells [73,74,75]. Other specific antimicrobial activities might include vesicle formation and the subsequent production of membrane holes in *E. coli* and *C. pseudotuberculosis*, intracellular ROS concentration increase in *S. aureus*, transcription inhibition in *S. aureus* and *E. coli*, and inclusion body formation and subsequent bacterial cell lysis in *S. pneumoniae* [75,76]. Furthermore, AuNPs interact with the negatively charged teichoic acids highly abundant within the cell wall of Gram-positive bacteria and the lipopolysaccharides present onto the outer membrane of Gram-negative bacteria [74]. It was observed that AuNPs exhibit higher antibacterial activity against Gram-negative bacteria, possibly due to a reduced cell wall thickness and more stable electrostatic interactions [73]. By binding to the proteins present onto the surface and further inhibiting their attachment to the receptors found within the host cells, AuNPs have also proven their efficiency against many viruses, including measles virus, Newcastle disease virus, respiratory syncytial virus, and chikungunya virus [74].

It has been observed that the antimicrobial activity is directly influenced by the size, functionalization, and concentration of AuNPs and by the microbial species and strain [75,77]. Specifically, a decrease in the average NP size and an increase in the concentration lead to improved antimicrobial effects [77]. Additionally, the nature and physico-chemical properties of the capping materials can also affect their activity, as they modulate the surface characteristics of the NPs [74]. However, the size, shape, dose, and surface functionalization of the NPs also have a direct impact on their cytotoxicity and biocompatibility. Therefore, a balance between the toxic effects and the antimicrobial activity must be ensured when designing AuNPs [78].

### 2.2. Silver Nanoparticles

Owing to their broad application in a variety of fields, including medicine, pharmacology, microbiology, cell biology, parasitology, chemistry, food technology, water purification, and house appliances, AgNPs are some of the most intensively studied among metal and metal oxide NPs [43,79,80,81]. They can be obtained through a wide range of methods, including the sol–gel method, hydrothermal method, thermal decomposition, chemical vapor deposition, microwave-assisted combustion, and biogenic synthesis methods. Their production involves reducing Ag^+^ to Ag^0^ using various biomolecules as electron donors, i.e., aldehydes, ketones, carboxylic acids, flavonoids, tannins, phenols, and proteins [79].

While their efficiency has been proven against more than 650 microorganisms, including Gram-positive and Gram-negative bacteria, fungi, and viruses, the precise mechanism of action of AgNPs is still not completely elucidated [82,83]. The primary mechanism is based on the interaction between the positively charged AgNPs and the negatively charged plasma cell membranes, which further results in the accumulation within the membrane and the consequent structural modifications and permeabilization due to cis-trans isomerization of the unsaturated membrane fatty acids [73,80,82,84]. In this manner, Ag^+^ is released from the outer surface of the NP, thus interacting with nucleic acids and proteins and further generating high amounts of ROS, namely, singlet oxygen, hypochlorous acid, hydroxyl radical, superoxide anion, and hydrogen peroxide [73,80,82,83]. Additionally, Ag^+^ can also bind to thiol groups within enzymes, forming stable Ag-S complexes that will modify the enzymatic configuration and block the activity site. In this manner, the enzyme is unable to perform its functions, thus causing cell or organism death [82]. Similar to AuNPs, AgNPs have proven higher efficiency towards Gram-negative bacteria due to a reduced cell wall thickness and an increased number of negative charges [73,84]. In the case of fungi, AgNPs interfere with the cell metabolism through the generation of ROS and modification of ergosterol levels and lead to cell lysis. Additionally, AgNPs are capable of inactivating viruses through the interaction with surface receptors and the consequent blocking of the viral entry phase, e.g., the sulfur groups of gp120 protein spikes onto the membrane of the human immunodeficiency virus or the viral envelope glycoproteins onto the herpes simplex virus type 1 through sulfonate groups [83,84].

The morphology of AgNPs in terms of size and shape is the main factor that affects the physico-chemical properties and the Ag^+^ release kinetics [79,83]. Thereby, studies have demonstrated higher antimicrobial bioactivity related to higher amounts of apoptotic agents, necrotic factors, and ROS for NPs with reduced sizes and larger surface areas [73,84]. Moreover, surface characteristics and the presence of coatings onto the NPs could also influence antimicrobial effects. Specifically, since particle aggregation results in the loss of bioactivity, surface coating using polymers or capping agents could offer anti-aggregating capacities [83,85].

However, high concentrations of silver ions could lead to toxic effects towards human cells. Therefore, the administration of AgNPs should avoid triggering the defense mechanisms of the host cells towards the NPs and the consequent adverse reactions or inhibition of their bioactivity [80,84,85].

### 2.3. Copper Nanoparticles

CuNPs are naturally synthesized by plants by reducing Cu^+^ and Cu^3+^ ions, acting as regulators of many important biological reactions, including electron transport chain, enzyme cofactors, and hormone signaling [86,87,88,89,90]. They can also be obtained by artificial methods, namely, physical processes that require sophisticated and expensive equipment and technology and chemical techniques, such as chemical or sonochemical reduction, thermal decomposition, electrochemical synthesis, hydrothermal processes, or microemulsions, which are more advantageous due to facile control, simple operation, limited equipment requirements, and high-quality particles [91,92]. However, as copper is highly sensitive to air, CuNP synthesis requires non-aqueous media and an inert atmosphere, such as nitrogen or argon, in order to avoid the formation of an oxide layer onto the surface [91,92,93]. Other strategies involve the protection of the NPs with capping agents or the conversion of CuNPs to copper oxide NPs (CuO NPs). However, capping agents are not capable of solving the oxidation issue completely, and the formation of CuO NPs leads to a decrease in their antimicrobial bioactivity [60,92,93]. Furthermore, their synthesis must consider the control of morphology and surface characteristics that will further influence their properties [91].

Generally, CuNPs act as oxidizing agents and are characterized by catalytic, sensing, electrical conductivity, and antimicrobial properties. Therefore, they are widely used as catalysts, nanowires, nanosensors, electron emitters, nanoprobes, and antibacterial, antifungal, and antiviral systems [87,91,92,94]. Additionally, since they are cheaper and involve low production costs, CuNPs are a potential competitor to AuNPs and AgNPs [87,91,92,95,96].

There are multiple mechanistic pathways involved in the antimicrobial bioactivity of CuNPs. First, copper acts on a similar principle to silver, as it combines with the thiol groups of key microbial enzymes and inactivates their functions [97]. Second, the Cu^+^ ion from cuprous oxide (Cu_2_O) forms complexes with the peptides found within the microbial membranes [98]. Third, the dissociation of Cu^2+^ from cupric oxide (CuO) induces ROS generation, leading to disturbances in amino acid and nucleic acid biosynthesis and the associated biochemical processes, e.g., iron displacement from iron–sulfur clusters and zinc or other metal ions competition for protein binding sites, disruption of the microbial membrane, and blocking of cellular respiration [60,95,96,98,99]. Additionally, it has been proven that CuNPs have a higher affinity towards amine and carboxyl groups onto the surface of the microbial cell [95,96]. Thus, they exhibit higher antimicrobial activities against *B. subtilis* and *E. coli* and silver-resistant species, such as *M. morganii* and *M. psychrotolerans* [86,95,96,97,98]. Furthermore, owing to their large surface-to-volume ratio, CuNPs have proven effective against plant pathogenic fungi, such as *F. oxysporum*, *C. lunata*, *A. alternata*, and *P. destructive* [98]. However, the medical use of CuNPs must take into account the possible cyto- and genotoxic effects [100].

### 2.4. Zinc Oxide Nanoparticles

Zinc is an essential trace mineral in the organism, playing vital roles in various physiological functions, such as enzyme activation for protein and nucleic acid synthesis and digestion, antioxidative processes, blood clotting, and bone metabolism [101,102,103]. Similarly, ZnO NPs are highly compatible with human cells, and their properties make them suitable for many biomedical applications, such as tissue engineering, drug delivery systems, antimicrobial coatings, bioimaging, and antioxidant agents. ZnO NPs can be synthesized through various methods, including thermal decomposition, combustion, vapor transport, the sol–gel method, the hydrothermal method, co-precipitation, ultrasonication, and green synthesis using plant extracts or microorganisms [101,104].

Under physiological conditions, ZnO NPs are highly stable; however, at slightly acidic pH, they undergo a rapid dissolution into Zn^2+^ ions [101,105]. In this context, ZnO NPs have proven to hold considerable potential as antimicrobial agents by exhibiting antibacterial, antifungal, and antiviral properties owing to their large surface area, reduced size, high surface reactivity, and ability to absorb UV radiation [102,104]. There are three main mechanisms involved in the antimicrobial activity of ZnO NPs. Specifically, the positively charged ZnO NPs interact with the negatively charged microbial cell walls or membranes through electrostatic forces. Subsequently, ZnO NPs attach onto the surface and distort the membrane structure, further leading to the internalization of the NPs within the cell and the loss of cell integrity, leakage of the intracellular components, and cell death [102,105,106,107,108,109]. On one hand, once internalized, NPs will release Zn^2+^ ions, which will interfere with metabolic and enzymatic processes and induce cell death [102,105,106,108,109]. On the other hand, NPs will generate ROS, such as superoxide anion, hydroxyl ion, and hydrogen peroxide, from their surface, causing oxidative stress by lipid peroxidation, DNA replication disruption and DNA damage, energy metabolism and cellular respiration inhibition, slow leakage of RNA, and rapid leakage of K^+^ ions [102,105,106,107,108,109]. Similarly, ZnO NPs enter fungal cells through diffusion and endocytosis, where they hinder mitochondrial functioning and cause irreversible nucleic acid and chromosome damages through Zn^2+^ release and ROS production [108].

The release of Zn^2+^ ions depends on the physico-chemical and morphological properties of the NPs [105,106,109]. Specifically, a particle size decrease results in superior antimicrobial properties [106], while rod-like structures lead to reduced Zn^2+^ ion release when compared to spherical NPs [102]. Furthermore, antimicrobial features are also influenced by the microbial strain, the concentration of NPs, and the time of interaction [106].

### 2.5. Titanium Oxide Nanoparticles

TiO_2_ is an FDA-approved compound for food, drugs, cosmetics, and food packaging uses [110]. It exists in three main polymorphs, namely, anatase, rutile, and brookite [111]. Synthetic routes for TiO_2_ NPs synthesis include the sol–gel method, hydrothermal and solvothermal methods, precipitation, and electrochemical processes, using titanium chloride, titanium isopropoxide, or titanyl sulfate-based compounds as precursors [111,112,113]. However, as these techniques are disadvantageous in terms of reaction time and particle size control, green synthesis methods have received increasing attention owing to their lack of toxicity and low costs [88,112].

Similar to ZnO NPs, TiO_2_ NPs have a wide band gap of 3.2 eV that can trigger the production of high-energy electron–hole pairs when exposed to UV light with wavelengths of 385 nm or lower [114,115]. Consequently, UV light irradiation leads to ROS production with high oxidative potential in the presence of oxygen [115,116]. As a result, ROS will cause DNA synthesis alteration, DNA and protein damage, and metabolic enzyme inactivation [114,117,118]. Other mechanisms include the attachment of the NPs to the microbial cell wall and subsequent internalization, thus damaging the integrity of the membrane and resulting in cell death [117,118]. Additionally, the accumulation of NPs onto the surface leads to the development of “pits” and the buildup of free radicals [118]. The antibacterial properties of TiO_2_ NPs have been reported against *E. coli*, *P. aeruginosa*, *S. aureus*, *L. monocytogenes*, *S. choleraesuis*, *V. parahaemolyticus*, *D. actinidiae*, and *P. expansum* [110].

The main factors influencing the antimicrobial properties of TiO_2_ NPs are related to their morphology, size, crystal structure, and surface charge and chemistry, as well as their concentration and exposure time [114,115,117,119]. Additionally, the difference in the composition of the cell wall in Gram-positive and Gram-negative bacteria might also affect their antimicrobial bioactivity [118].

### 2.6. Magnesium Oxide Nanoparticles

MgO NPs have also been recognized by the FDA as safe materials [120], which has attracted scientific interest towards their application in biomedical areas. MgO NPs are non-toxic and easy to obtain, exhibiting antimicrobial properties against Gram-positive and Gram-negative bacteria, fungi, and viruses and biofilm-inhibiting features [120,121,122]. MgO comprises a lattice of Mg^2+^ and O^2-^ ions held by ionic bonds [123]. MgO NPs can be synthesized through a variety of methods, including combustion, calcination, sol–gel, hydrothermal method, co-oxidation, and wet precipitation [101,123,124,125]. However, current works focus on the use of green methods in order to generate NPs with lower toxicity [126].

Studies have reported that the underlying mechanisms for the antimicrobial properties of MgO NPs are based on the dissociation of Mg^2+^ ions in elevated pH values characteristic for bacterial and yeast cultures [127]. Additionally, they generate the superoxide anion through the reaction with oxygen present on the microbial cell surface. Further, the ROS will induce lipid peroxidation and protein and phospholipid damages that will disrupt the membrane and cause cell death [124,127,128,129,130]. Other mechanisms could also be involved, such as quorum sensing disruption due to the surface area, chemistry, roughness, and wettability of the NPs [128,130]. MgO NPs have proven their antibacterial properties against *S. aureus*, *P. aeruginosa*, and *E. coli* [120,131] and inhibited the formation of *E. coli*, *K. pneumoniae*, and *S. aureus* biofilms [122].

In addition to the influence of NP size; shape; composition; and surface properties, such as hydrophobicity, the antimicrobial properties of MgO NPs are also affected by the microbial species and strain (e.g., higher bioactivity against Gram-positive than Gram-negative bacteria), the concentration of the NPs, and the time of exposure [122,123,127,128].

### 2.7. Iron Oxide Nanoparticles

Owing to their superparamagnetic and high magnetic susceptibility, Fe_3_O_4_ NPs have attracted great scientific interest for their application within the biomedical field, such as drug delivery systems, bioimaging, and theranostics [132]. Since their behavior is strongly dependent upon their size, shape, structure, surface chemistry, and colloidal stability, the choice of synthesis method is highly important. There are three main routes for Fe_3_O_4_ NPs synthesis, namely, physical, chemical, and biological techniques, but the most commonly applied method is chemical co-precipitation [133,134,135,136]. Nonetheless, researchers are currently focusing on green synthesis methods, as the so-obtained NPs are less toxic, more stable, and have reduced sizes and agglomeration tendency [137].

Similarly, the small size and high surface area allow Fe_3_O_4_ NPs to adhere to the bacterial cell wall through electrostatic and intermolecular forces, causing membrane depolarization and structure integrity loss. Additionally, NPs diffuse through the membrane, interacting with membrane lipids and proteins and changing the osmotic pressure. As a result, there is a leakage of the intracellular content and a shrinkage of the cell that will lead to microbial cell death [65,129,138,139]. Furthermore, Fe^2+^ and Fe^3+^ ions are released from the NPs, leading to the production of high amounts of ROS and consequent DNA replication disruption, DNA double-strand breaking, and lipid peroxidation [129,138,139,140]. The antibacterial properties of Fe_3_O_4_ NPs have been proven against *E. coli*, *K. pneumoniae*, *P. aeruginosa*, *B. subtilis*, *S. epidermidis*, and *H. pylori* [137,138,139]. However, results have demonstrated increased bactericidal effects against Gram-negative bacteria compared to Gram-positive bacteria [129].

## 3. Inorganic Nanoparticle-Based Composite Films for Antimicrobial Applications

**Figure 6:**
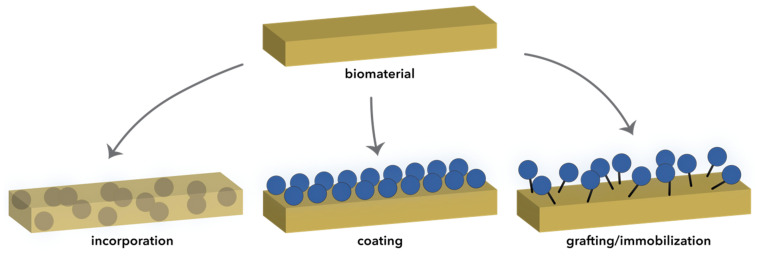
Schematic representation of the main strategies for obtaining composite materials based on inorganic NPs and organic/inorganic films.

There are three main approaches for achieving film-based composite materials, namely, through the incorporation of the inorganic NPs into the matrix, coating of the film with inorganic NPs, or grafting/immobilization of the inorganic NPs onto the surface of the film (Figure 6) [27,141]. In this manner, the properties of both phases are enhanced, which allows for their use in applications such as implant or catheter coatings and wound dressings.

In this context, the studies discussed below target the application of composite films comprising inorganic NPs and an organic/inorganic matrix against microbial infections [142]. The criteria involved in the process of article selection involved papers published after 2018 from the Scopus database using the keywords “composite film”, “antimicrobial”, and “gold nanoparticles”, “silver nanoparticles”, “copper nanoparticles”, “zinc oxide nanoparticles”, “titanium oxide nanoparticles”, “magnesium oxide nanoparticles”, or “iron oxide nanoparticles”. Studies focusing on their applications for food and cosmetic industries or water and soil purification were not considered. Therefore, 38 relevant studies were identified and categorized based on the type of NP and film material used (Table 2).

For instance, Zhu et al. developed AuNPs and silk fibroin-based composite films by mixing the 4,6-diamino-2-pyrimidinethiol-functionalized AuNPs into the silk fibroin solution. After the evaporation of the solvent, the mixed-matrix membranes were tested against *E. coli* and multidrug-resistant *E. coli*, both in vitro and in vivo, using an *E. coli*-infected rat wound model [143].

On one hand, natural polymers represent the material of choice when designing composite films for biomedical applications owing to their increased biocompatibility and biomimicry. In this context, Gromovykh et al. investigated the antibacterial and antifungal activity and cytotoxicity of AgNPs impregnated within a bacterial cellulose film. While the nanocomposite system exhibited antibacterial properties against *S. aureus*, *B. coagulans*, and *E. coli*, results proved no fungicidal character. Additionally, the antitumor effects of the composite films demonstrated a potential towards their further applications as scaffolds for cancer treatment [144]. Similarly, Chatchawanwirote et al. developed silver nanoprisms through a light-induced transformation reaction of silver colloids that were further impregnated within a bacterial cellulose film. Results regarding antibacterial activity and lack of toxicity against human dermal fibroblasts confirmed these films’ potential for wound dressing applications [145]. Another study by Khamrai et al. focused on obtaining a mussel mimetic, antibacterial wound healing transdermal patch system by grafting dopamine onto the surface of carboxymethylated bacterial cellulose through an amidation reaction and further reducing Ag^+^ and graphene oxide to obtain composite films. The composite films proved bactericidal properties and biocompatibility towards fibroblast cells and human lung epithelial cells, as well as the potential for wound healing owing to the acceleration of cell proliferation and migration [146]. Additionally, Limaye et al. obtained cellulose nanofiber and polyvinyl alcohol-based films impregnated with AgNPs for antibacterial applications against *B. subtilis* and *E. coli* bacterial strains [147]. Furthermore, Wang et al. investigated the antimicrobial bioactivity of silk sericin/agar films containing AgNPs against *S. aureus* and *E. coli* bacteria. In addition to their suitable antibacterial properties, the AgNP containing polymeric films exhibited excellent hydrophilicity and good mechanical properties, making them potential candidates for the development of wound dressings or tissue engineering scaffolds [148]. Liu et al. developed similar polymeric films, with an additional step of polyelectrolyte membrane coating through the layer-by-layer synthesis of polyacrylic acid/poly (dimethyldiallylammonium chloride)/polyacrylic acid onto the surface of the silk sericin/agar film [149]. Similarly, the same group later developed a polydopamine-treated silk sericin/agar film that could assist the synthesis of high-density AgNPs onto the surface of the film [150]. Another study also synthesized polydopamine-treated silk sericin/agar films for the successful immobilization of AgNPs. However, their study involved a supplementary step, namely, the addition of an intermediary layer of ZnO that was also coated with polydopamine, thus improving the antibacterial properties of the resulting composite film [151]. Moreover, Sionkowska et al. developed collagen/chitosan films containing AgNPs with potential bacteriostatic activity and suitable mechanical properties for wound dressing applications [152]. Another study by Zhu et al. investigated the effects of a composite system consisting of nacre-like konjac glucomannan-montmorillonite composite films and AgNPs incorporated into the layered structure against *S. aureus* and *E. coli* bacteria. Results proved suitable antibacterial properties, as well as good biocompatibility towards RAW264.7 cells [153].

On the other hand, considering their suitable mechanical properties and ease of processability, synthetic polymers also hold potential in designing composite films for antimicrobial therapies. In this regard, Olmos et al. developed low-density polyethylene and AgNP-based composite films by obtaining a well-dispersed powder using high-energy ball milling, followed by powder hot pressing. In vitro tests against *E. coli* cultures revealed that an increase in AgNP content from 0.5% to 2% led to a decrease in the amounts of microorganisms and extracellular polymeric substances which are associated with biofilm development [154]. Similarly, Li et al. investigated the potential of metallocene polyethylene containing AgNPs as coatings for medical devices. Antibacterial and silver ion release results showed excellent bactericidal properties against *S. aureus* and *E. coli* and a slow ion release for more than 30 days [155]. Another study by Lethongkam et al. developed an endotracheal tube coating comprising a polyamide matrix and dispersed AgNPs. The obtained film exhibited a broad antimicrobial activity against both planktonic growth and microbial adhesion, thus providing a potential strategy against ventilator-associated pneumonia [156]. Moreover, Hoş et al. investigated the potential of a composite film consisting of polycaprolactone and AgNP-coated hydroxyapatite for implant applications. The presence of AgNPs ensured the antibacterial activity against *S. aureus*, *S. epidermidis*, and *E. coli*, while the hydroxyapatite component could ensure an enhanced implant acceptance and bone regeneration capacities [157]. The antimicrobial character of AgNPs and silicone hydrogel-based composite films against *S. aureus*, *P. aeruginosa*, and *E. coli* was also investigated by Mourad et al. The composite films proved their potential in contact lens applications [158]. Furthermore, Cruz-Pacheco et al. coated polyetheretherketone with one or two layers of AgNPs to inhibit *E. coli*, *S. marcescens*, and *B. licheniformis* growth for potential biomedical applications [159]. Additionally, López-Saucedo et al. developed polytetrafluorethylene films grafted with methyl methacrylate and subsequently with N-vinylimidazole using gamma-rays that were used for the immobilization of AgNPs. The composite films were tested against *S. aureus* and *E. coli*, thus proving their antibacterial properties [160]. Anancharoenwong et al. performed a comparative study between AgNPs, TiO_2_ NPs, and benzoic acid-containing polyurethane composite films against *P. aeruginosa* and *S. aureus* strains. Although results showed the strongest antimicrobial behavior for the AgNP-based composite films, the thermal stability of the polyurethane films decreased with the addition of fillers in all samples [161]. Similarly, Jamróz et al. compared the antibacterial properties of AgNPs and selenium NPs incorporated into furcellaran-gelatin films against *S. aureus*, multidrug-resistant *S. aureus*, and *E. coli*. Results demonstrated an enhanced antibacterial behavior of selenium NP-containing composite films at all concentrations, in contrast to AgNP-based composite films, which only exhibited bactericidal properties against *E. coli* at the highest concentration [162]. Another study by Jatoi et al. developed electrospun polyacrylonitrile nanofibers containing AgNP-coated titanium dioxide. Antimicrobial tests against *S. aureus* and *E. coli* strains revealed that an increase in NP concentration led to enhanced antibacterial properties [163]. Additionally, Maharubin et al. investigated the bactericidal effects of AgNPs covalently bonded on the surface of polyvinyl chloride films followed by the self-assembly of radiating acicular structured ZnO nanowires. The potential of these composite films for urinary catheter surface modification was demonstrated against *S. aureus* [164].

AgNPs can also be used in the synthesis of inorganic material-based composite films. Specifically, Zhu et al. obtained antibacterial nanocomposite films using graphene oxide nanosheets that were further decorated with triangular AgNPs. While the results proved antibacterial properties against *S. aureus* and *E. coli* cultures, further studies are necessary in order to confirm the lack of toxicity of the composite systems for biomedical applications [165].

Furthermore, Sivaranjana et al. developed cellulose films that were dipped in *Cassia alata* leaf extract solutions for the subsequent in situ reduction of copper sulfate for the generation of NPs within the matrix. In addition to a good antibacterial activity against *E. coli* cultures, the presence of CuNPs improved the mechanical properties of the composite film in terms of tensile strength, proving its suitability for medical applications [166]. Another study investigated the effects of CuNP incorporation into polyvinyl chloride films on their antibacterial, thermal, and rheological properties. The presence of the NPs improved the thermal stability and the processability of the polymer and ensured antibacterial bioactivities against *E. coli* cultures [167]. Moreover, Kruk et al. synthesized polyelectrolyte-copper nanocomposite coatings consisting of poly(diallyldimethylammonium chloride) as a polycation, poly(sodium 4-styrenesulfonate) as a polyanion, and negatively charged CuNPs through a layer-by-layer method. The antibacterial character of the polyelectrolyte thin films proved their potential as medical device coatings for the prevention of microbial surface contamination [168]. In addition, Jayaramudu et al. compared the efficiency of CuNPs and CuO NP-containing chitosan films against *B. subtilis* and *E. coli* bacterial strains. While both types of composite films exhibited excellent antibacterial properties, CuO NP-containing chitosan films showed stronger bioactivities [169]. Moreover, Muñoz-Escobar et al. developed electrospun polycaprolactone nanofiber-based films containing CuO NPs, proving their efficiency against a broad spectrum of clinically important bacterial strains, including *S. mutans*, *K. oxytoca*, *S. aureus*, *P. aeruginosa*, *B. subtilis*, and *E. coli* [170].

ZnO NPs have also been used for the design and fabrication of composite films for biomedical antimicrobial therapies. For instance, Hezma et al. developed antimicrobial composite films comprising chitosan and polyvinyl alcohol polymeric blends and ZnO NPs. Antimicrobial assays against *S. aureus*, *E. coli*, *C. albicans*, and *A. niger* proved that the incorporation of ZnO NPs significantly enhanced their antimicrobial efficacy. Additionally, the composite films exhibited good thermal stability and mechanical strength [171]. Similarly, Ai et al. investigated the antimicrobial properties of silk sericin and polyvinyl alcohol polymeric films coated with polydopamine for the successful immobilization of ZnO NPs. Results proved suitable antibacterial features and enhanced mechanical performance [172]. Another study by Jayakumar et al. focused on the development of polyvinyl alcohol films containing ZnO NPs and lipopeptides for enhanced antimicrobial properties. While the presence of ZnO NPs slightly reduced microbial colonization, the incorporation of lipopeptides led to a significant increase in the antimicrobial properties of the composite film [173]. Furthermore, Liu et al. obtained composite films based on thermal-responsive shape memory polyurethane and ZnO NPs that could allow for adhered biofilm detachment and residual bacteria elimination. Antibacterial assays against *S. aureus* strain proved both bactericidal and biofilm detachment properties [174]. Moreover, Al-Jumaili et al. focused on another approach for the synthesis of ZnO NP-based composite films. Specifically, they combined the simultaneous plasma polymerization of the geranium essential oil and the thermal decomposition of zinc acetylacetonate for the single-step fabrication of the composite film. Results proved the release of ZnO NPs that could be further modulated through a bilayer structure and the antibacterial activity against both Gram-positive and Gram-negative bacteria [175].

TiO_2_ NPs were used in the fabrication of chitosan-based composite films in a study performed by Hussein et al. Results proved that the addition of inorganic NPs into the polymeric films prevented the bacterial proliferation of both Gram-positive and Gram-negative bacteria [176]. Furthermore, Qu et al. investigated the antibacterial properties of TiO_2_ NP-containing zein and chitosan films against *S. enteritidis*, *S. aureus*, and *E. coli*. Composite film characterization demonstrated improved mechanical properties, thermal stability, and hydrophobicity and enhanced antibacterial activity when compared to the unmodified film. Additionally, UV light irradiation further increased antibacterial properties by 26.44%, 21.45%, and 21.78%, respectively [177].

Furthermore, Hussein et al. performed a comparative study between MgO NP-containing polyvinylidene fluoride films obtained either by electrospinning or by spin coating. Microbiological assays showed that only electrospun films exhibited antibacterial properties, possibly due to the presence of the NPs on the surface of the nanofibers [178].

Additionally, Dacrory et al. developed Fe_3_O_4_ NPs and cyanoethyl cellulose-based composite films and tested their antimicrobial features. Results proved their potential in antimicrobial therapies, as the composite films inhibited the proliferation of all microbial species [179].

## 4. Conclusions and Future Perspectives

Drug-resistant microorganisms are becoming a critical health issue, as there are up to 700,000 deaths due to antimicrobial resistance worldwide annually, with the number expected to rise to 10 million deaths by 2050. The need for designing novel treatment strategies for counteracting this issue has led to a rise in antimicrobial NPs. Specifically, numerous studies are investigating the antimicrobial properties and the associated mechanisms of metal and metal oxide NPs, such as gold, silver, copper, zinc oxide, titanium oxide, magnesium oxide, or iron oxide. In this context, the number of research articles focusing on these types of NPs for antimicrobial therapies has doubled since 2015. While most of the underlying mechanisms involve microbial membrane disruption, ion release, and ROS generation, multiple pathways are specific to each type of NP. Therefore, a balance between the antimicrobial efficiency and the production costs, processability, and cytotoxicity must be taken into consideration when selecting the therapeutic NPs. Moreover, in order to direct their use towards more specific applications, including medical device coatings or wound dressings, these NPs have been incorporated or attached onto the surface of organic/inorganic films. In this manner, composite films with enhanced antimicrobial behaviors and suitable mechanical properties can be developed. The types of matrices used for such applications comprise various materials, ranging from natural or synthetic polymers to carbonaceous materials, such as graphene. While there are many studies focusing on the bactericidal and fungicidal properties of these composite films, the attention should also shift towards their antiviral properties, especially due to the present pandemic. In this context, these materials could be used for the fabrication of medical devices and textiles that would prevent viral contamination or spreading.

## Figures and Tables

**Table 1:** Summary of the main synthesis methods and antimicrobial mechanisms for AuNPs, AgNPs, CuNPs, ZnO NPs, TiO_2_ NPs, MgO NPs, and Fe_3_O_4_ NPs.

NP Type	Synthesis Methods	Antimicrobial Mechanisms
AuNPs	chemical reduction and green synthesis methods	- Interactions between NPs and the microbial cell wall through electrostatic forces and carbohydrate, lipid, and protein binding; - Damages of the microbial cell membrane and wall; - Impairment of ribosome and mitochondrion functions; - Inhibition of thiol groups present within bacterial cells; - Formation of vesicles and production of membrane holes; - Intracellular ROS production; - Inhibition of transcription; - Inclusion body formation and subsequent bacterial cell lysis.
AgNPs	chemical reduction, sol–gel method, hydrothermal method, thermal decomposition, chemical vapor deposition, microwave-assisted combustion, and biogenic synthesis methods	- Interactions between NPs and the plasma cell membranes; - Accumulation within the cell membrane and consequent structural modifications and permeabilization due to cis-trans isomerization of the unsaturated membrane fatty acids; - Silver ion release and consequent generation of ROS; - Binding to thiol groups within enzymes and formation of stable Ag-S complexes that will modify the enzymatic configuration and block the activity site; - Modification of ergosterol levels in fungi; - Interactions with surface receptors and consequent blocking of viral entry phases.
CuNPs	physical methods, chemical or sonochemical reduction, thermal decomposition, electrochemical synthesis, hydrothermal processes, or microemulsions, and green synthesis methods	- Binding to thiol groups within enzymes and formation of stable Ag-S complexes that will modify the enzymatic configuration and block the activity site; - Release of copper ions and formation of complexes with the peptides found within the microbial membranes; - ROS production and disturbances in amino acid and nucleic acid biosynthesis and the associated biochemical processes (iron displacement from iron–sulfur clusters and zinc or other metal ions competition for protein binding sites, disruption of the microbial membrane, and blocking of cellular respiration).
ZnO NPs	thermal decomposition, combustion, vapor transport, sol–gel method, hydrothermal method, co-precipitation, ultrasonication, and green synthesis methods	- Interactions between NPs and the plasma cell membranes; - Attachment of NPs onto the surface and distortion of the membrane structure, further leading to the internalization of the NPs within the cell and the loss of cell integrity, leakage of the intracellular components, and cell death; - Dissolution of zinc ions at acidic pH, which will interfere with metabolic and enzymatic processes and induce cell death; - Production of ROS and subsequent lipid peroxidation, DNA replication disruption and DNA damage, energy metabolism and cellular respiration inhibition, slow leakage of RNA, and rapid leakage of K^+^ ions.
TiO_2_ NPs	sol–gel method, hydrothermal and solvothermal method, precipitation, electrochemical processes, and green synthesis methods	- ROS production under UV light irradiation and consequent DNA synthesis alteration, DNA and protein damage, and metabolic enzyme inactivation; - Attachment of NPs onto the surface and distortion of the membrane structure, further leading to the internalization of the NPs within the cell and the loss of cell integrity, leakage of the intracellular components, and cell death; - Development of “pits” and the buildup of free radicals due to NP accumulation.
MgO NPs	combustion, calcination, sol–gel, hydrothermal method, co-oxidation, wet precipitation, and green synthesis methods	- Dissociation of magnesium ions at elevated pH levels and consequent ROS production that will induce lipid peroxidation, protein and phospholipid damages, membrane disruption, and cell death; - Quorum sensing disruption due to the surface area, chemistry, roughness, and wettability of the NPs.
Fe_3_O_4_ NPs	physical, chemical, and biological methods	- Adherence of the NPs to the bacterial cell wall through electrostatic and intermolecular forces and consequent membrane depolarization and structure integrity loss; - Diffusion of NPs through the membrane, interacting with membrane lipids and proteins and changing the osmotic pressure; - Leakage of the intracellular content and shrinkage of the cell; - Release of iron ions and consequent production of high amounts of ROS and DNA replication disruption, DNA double-strand breaking, and lipid peroxidation.

**Table 2:** Summary of the identified studies investigating the antimicrobial properties of inorganic NP-containing composite films.

NP Type	NP Synthesis Method	NP Mean Size [nm]	Film Material Type	Film Synthesis Method	Microbial Species	Application	Ref.
AuNPs	chemical reduction	2.44	silk fibroin	solvent evaporation	*E. coli* multidrug-resistant *E. coli*	wound dressing	[143]
AgNPs	metal-vapor synthesis	8–12	bacterial cellulose	*Gluconacetobacter hansenii* cultivation	*S. aureus* acid-resistant *B. coagulans* *E. coli* *A. niger* *C. albicans*	medical material coatings	[144]
	chemical reduction followed by light-induced transformation reaction	31.62	bacterial cellulose	n.r.	*P. aeruginosa* *E. faecalis* methicillin-resistant *S. aureus* *E. coli*	wound dressing	[145]
	green reduction	15	bacterial cellulose	*Gluconacetobacter xylinus* cultivation	*S. aureus* *L. fusiformis* *E. coli* *P. aeruginosa*	wound dressing	[146]
	chemical reduction	20	cellulose nanofibers and polyvinyl alcohol	n.r.	*B. subtilis* *E. coli*	biological applications	[147]
	UV-assisted in situ reduction	20–80	silk sericin and agar	solvent evaporation	*S. aureus* *E. coli*	wound dressings and tissue engineering	[148]
	UV-assisted in situ reduction	50–80	silk sericin and agar	solvent evaporation	*S. aureus* *E. coli*	wound dressings, artificial skin, and tissue engineering	[149]
	in situ reduction	300–500	silk sericin and agar	solvent evaporation	*S. aureus* *E. coli*	wound dressings and tissue engineering	[150]
	in situ reduction	n.r.	silk sericin and agar	solvent evaporation	*S. aureus* *E. coli*	antibacterial coatings wound dressing tissue engineering	[151]
	chemical reduction	n.r.	collagen and chitosan	solvent evaporation	*S. aureus*	wound dressings	[152]
	in situ reduction	10–20	konjac glucomannan and montmorillonite	self-assembly and vacuum filtration	*S. aureus* *E. coli*	biomedical applications	[153]
	commercial AgNPs	50	low-density polyethylene	powder hot pressing	*E. coli*	biomedical applications	[154]
	commercial AgNPs	60–120	metallocene polyethylene	solvent evaporation	*S. aureus* *E. coli*	medical device coatings	[155]
	green reduction	60	polyamide	dip-coating	*S. aureus* methicillin-resistant *E. faecalis* *P. aeruginosa* carbapenem-resistant *K. pneumoniae* extended-spectrum b-lactamase-producing *K. pneumoniae* *A. baumannii* carbapenem-resistant *A. baumannii* *C. albicans*	endotracheal tube coating	[156]
	chemical reduction	0–30	polycaprolactone	polymer melting	*S. aureus* *S. epidermidis* *E. coli*	medical implants	[157]
	in situ reduction	2–100	silicone	mold injection	*S. aureus* *P. aeruginosa* *E. coli*	contact lenses	[158]
	chemical reduction	<30	polyetheretherketone	n.r.	*E. coli* *S. marcescens* *B. licheniformis*	biomedical applications	[159]
	chemical reduction	n.r.	polytetrafluorethylene	commercial films	*S. aureus* *E. coli*	medical device coatings	[160]
	chemical reduction	10	polyurethane	solvent evaporation	*P. aeruginosa* *S. aureus*	biomedical applications	[161]
	chemical reduction	n.r.	furcellaran and gelatin	solvent evaporation	*S. aureus* multidrug-resistant *S. aureus* *E. coli*	biomedical applications	[162]
	chemical reduction	5.9	polyacrylonitrile	electrospinning	*S. aureus* *E. coli*	biomedical applications	[163]
	commercial AgNPs	n.r.	polyvinyl chloride	solvent evaporation	*S. aureus*	urinary catheter coating	[164]
	photochemical reaction	49.3–114	graphene oxide	solvent evaporation	*S. aureus* *E. coli*	biomedical applications	[165]
CuNPs	in situ green reduction	60–69	cellulose	solvent evaporation	*E. coli*	biomedical applications	[166]
	commercial CuNPs	50	polyvinyl chloride resin	melt mixing	*E. coli*	medical device manufacturing	[167]
	chemical reduction	50–70	poly(diallyldimethylammonium chloride) and poly(sodium 4-styrenesulfonate)	layer-by-layer method	*S. aureus*	medical device coatings	[168]
	chemical reduction	7	chitosan	solvent evaporation	*B. subtilis* *E. coli*	biomedical applications	[169]
CuO NPs	chemical reduction	35	polycaprolactone	electrospinning	*S. mutans* *K. oxytoca* *S. aureus* *P. aeruginosa* *B. subtilis* *E. coli*	wound dressings	[170]
ZnO NPs	sol–gel method	n.r.	chitosan and polyvinyl alcohol	solution casting	*S. aureus* *E. coli* *C. albicans* *A. niger*	biomedical applications	[171]
	commercial ZnO NPs	60–120	silk sericin and polyvinyl alcohol	solvent evaporation	*S. aureus* *E. coli*	wound dressings	[172]
	commercial ZnO NPs	n.r.	polyvinyl alcohol	solvent evaporation	*S. aureus* *K. pneumoniae* *P. aeruginosa*	biomedical applications	[173]
	n.r.	n.r.	thermal-responsive shape memory polyurethanes	solution casting	*S. aureus*	antibiofilm platforms	[174]
	gas-phase NP nucleation	60–80	geranium essential oil	plasma polymerization	*S. aureus* *E. coli*	medical device and implant coatings	[175]
TiO_2_ NPs	commercial TiO_2_ NPs	<100	chitosan	solution casting	*B. cereus* *S. aureus* *E. coli*	biomedical applications	[176]
	sol–gel method	5.12–6.29	zein and chitosan	solvent evaporation	*S. enteritidis* *S. aureus* *E. coli*	biomedical applications	[177]
	commercial TiO_2_ NPs	21	polyurethane	solvent evaporation	*P. aeruginosa* *S. aureus*	biomedical applications	[161]
MgO NPs	commercial MgO NPs	30–40	polyvinylidene fluoride	electrospinning and spin coating	*S. aureus* *E. coli*	wound dressings	[178]
Fe_3_O_4_ NPs	in situ co-precipitation	>20	cyanoethyl cellulose	solvent evaporation	*S. aureus* *E. coli* *C. albicans* *A. niger*	biomedical applications	[179]

## Data Availability

Not applicable.
